# Mechanism and therapeutic potential of traditional Chinese medicine extracts in sepsis

**DOI:** 10.3389/fphar.2024.1365639

**Published:** 2024-07-03

**Authors:** Chen-Fei Fu, Jian-Long Li, Jia-Wei Chen, Hao Liang, Wen-Rui Zhao, Shi-Yu He, Xiao-Wei Ma, Xiao-Fan Yang, He-Lin Wang

**Affiliations:** ^1^ Heilongjiang University of Chinese Medicine, Harbin, China; ^2^ Liaoning Normal University, Dalian, China; ^3^ Guizhou University of Traditional Chinese Medicine, Guiyang, China; ^4^ Hongqi Hospital Affiliated to Mudanjiang Medical University, Mudanjiang, China; ^5^ Shenzhen Pingle Orthopedic Hospital, Shenzhen, China; ^6^ Donghuashi Community Health Service Center, Beijing, China

**Keywords:** sepsis, traditional Chinese medicine, extracts, pharmacological action, mechanism

## Abstract

Sepsis is a complex syndrome characterized by multi-organ dysfunction, due to the presence of harmful microorganisms in blood which could cause mortality. Complications associated with sepsis involve multiple organ dysfunction. The pathogenesis of sepsis remains intricate, with limited treatment options and high mortality rates. Traditional Chinese medicine (TCM) has consistently demonstrated to have a potential on various disease management. Its complements include reduction of oxidative stress, inhibiting inflammatory pathways, regulating immune responses, and improving microcirculation. Traditional Chinese medicine can mitigate or even treat sepsis in a human system. This review examines progress on the use of TCM extracts for treating sepsis through different pharmacological action and its mechanisms. The potential targets of TCM extracts and active ingredients for the treatment of sepsis and its complications have been elucidated through molecular biology research, network pharmacology prediction, molecular docking analysis, and visualization analysis. Our aim is to provide a theoretical basis and empirical support for utilizing TCM in the treatment of sepsis and its complications while also serving as a reference for future research and development of sepsis drugs.

## 1 Introduction

The management of sepsis has emerged as a critical scientific challenge, necessitating urgent attention as a significant global public health crisis (Groote and Meersch-Dini, 2022). The extensive heterogeneity observed in host, pathogen, and the environment contributes significantly to disease development (Jones et al., 2021). Despite recent advancements in sepsis treatment and advanced life support, mortality rate from septic shock remains approximately is 50% (Vincent et al., 2019). The ongoing pursuit of improved treatment resulted in research for development of alternative therapies to manage sepsis.

In traditional Chinese medicine, sepsis is commonly classified as one of the exogenous febrile diseases documented in ancient Chinese medical literature, including the Treatise on Febrile Diseases, Treatise on Epidemic Febrile Diseases, and other relevant publications (Fan et al., 2020). The theory of traditional Chinese medicine (TCM) emphasizes that the treatment of sepsis should focus on clearing heat and detoxification, restoring normal bowel movement, promoting blood circulation and removing blood stasis, as well as strengthening immune system. According to the concept of entirety and the method of treatment with syndrome differentiation of TCM, the clinical efficacy of sepsis was significantly improved and the case fatality rate effectively reduced (Zhao et al., 2017). The collection, processing, and preparation of Chinese herbal medicine adhere to the fundamental principles of TCM, which boasts into a rich history for both theory and practice when it comes to treating critically ill patients. The practitioners of TCM have expertise for identification of symptoms related to acute and critical illnesses, as well as formulating appropriate TCM treatments. Traditional Chinese medicine can serve as an adjunctive therapy in the management of sepsis due to its advantageous characteristics, including a complex composition, multiple targets of action, and involvement in various signaling pathways (Usmani et al., 2021). It exhibits diverse effects such as anti-inflammatory properties, improvement of microcirculation, alleviation of gastrointestinal dysfunction, and enhancement of the immune system. Moreover, it may even confer protection against organ damage caused by sepsis (Usmani et al., 2021). The efficacy and safety of employing this evidence-based TCM treatment in conjunction with intensive sepsis therapy increasingly acknowledged by the medical community and patients. Traditional Chinese medicine has played a crucial role in the management of COVID-19 patients, providing us novel insights into the merits and significance of employing traditional Chinese medicine for sepsis treatment (Lyu et al., 2021). Traditional Chinese medicine has been incorporated into the standard treatment for sepsis (Wang X.-H. et al., 2022), including medications such as Xuebijing injection (XBJ), Shenfu injection, Qingwen Baidu decoction, Xuanbai Chengqi decoction (XBCQ), and Qingmai decoction (QWBD). The significant advancements have been made in the exploration of the efficacy and mechanism of action for TCM to sepsis treatment, with an increasing number of studies corroborating the prominent role in the prevention and management of sepsis (Zheng et al., 2023). Although there have been review articles discussing the research progress of traditional Chinese medicine compounds and monomer extracts in the treatment of sepsis and multi-organ injury (Song et al., 2023), this review merely presents a superficial list of various monomer extracts used for sepsis treatment, lacking a comprehensive analysis and discussion on their therapeutic mechanisms. It is noteworthy that our review commences with an exploration of the etiology and pathogenesis of sepsis, subsequently delving into the therapeutic strategies for sepsis utilizing traditional Chinese medicine extracts based on their respective mechanisms of action. This approach facilitates a comprehensive understanding of TCM’s therapeutic process for sepsis, as TCM typically addresses diseases through multiple pathways and targets. Categorizing interventions based on their mechanism rather than solely relying on TCM extracts enhances our comprehension and cognition of TCM’s efficacy in treating sepsis. Our review establishes correlations between the mechanisms of traditional Chinese medicine extracts for treating sepsis and its pathogenesis, thereby emphasizing the logical basis behind TCM disease management and bolstering its persuasiveness. Consequently, this review serves as a valuable reference for future development of sepsis medications.

## 2 Overeview of sepsis

Sepsis is a life-threatening syndrome of multiple organ dysfunction caused by dysregulation in the body’s immune response to infection (Sygitowicz and Sitkiewicz, 2021). The mortality rate of patients with sepsis can exceed 30%–35% in the absence of timely and effective intervention, as a result of an increased inflammatory response caused by immunosuppression or primary infection (Vincent et al., 2019). The complications induced by sepsis involve multi-organ dysfunction such as pulmonary impairment, cerebral injury, renal impairment, hepatic impairment, and myocardial damage (Sun et al., 2020; Zhou and Liao, 2021; Chang et al., 2022; Sekino et al., 2022; Zhao et al., 2022). The high mortality rate resulting from sepsis is primarily attributed to the occurrence of multi-organ failure induced by the disease. The pathogenesis of sepsis is highly intricate at the cellular and molecular level, which encompasses various pathophysiologic processes such as inflammation imbalance, immune dysfunction, mitochondrial damage, coagulation disorders, neuroendocrine immune network abnormalities, endoplasmic reticulum stress, and autophagy, ultimately resulting in multi-organ dysfunction (Huang et al., 2019a). According to the conventional perspective, following an initial phase of excessive inflammation, a subsequent phase of diminished inflammation, partially attributed to the release of anti-inflammatory cytokines, leads to profound immunosuppression (Sheth et al., 2019). Currently, preliminary results indicated that the stages of inflammation promotion and immune suppression may occur concurrently (Zhong and Yin, 2023), rendering the interaction between host and pathogen conducive for disease development. The high-risk factors for sepsis patients encompass advanced age, gender, presence of immunosuppressive diseases, medications, history of cancer, diabetes mellitus, alcohol misuse, and the utilization of indwelling urinary catheters (Tokuda et al., 2023). The severity of sepsis depends on the host’s age, comorbidities, and immune status, as well as pathogenic factors such as toxicity, microbial species, and infection burden (Olinder et al., 2022). In addition to pathogen-related factors, there may also be host genetic factors that could increase the risk of developing sepsis (Mukherjee et al., 2019). The latest guidelines released by the “Surviving Sepsis Campaign” in October 2021 further emphasize the implementation of sepsis screening for standardized treatment, enhance infection control and optimize antimicrobial drug utilization, as well as recommend novel treatment techniques and concepts. The current international clinical guidelines primarily include rapid screening and early diagnosis, accurate identification and control of infection sources and pathogenic microorganisms, timely and effective antibiotic treatment and fluid resuscitation, management of hemodynamics, and mechanical ventilation (Fitridge and Thompson, 2007).

Despite some advancements in the research and treatment of sepsis in recent years, a comprehensive understanding of its pathogenesis and key mechanisms is still lacking, necessitating further improvements in targeted therapies. However, lack of effective treatment methods hinders the management of cellular and tissue organ damage resulted from sepsis. The treatment of sepsis remains a global challenge, involving the prevention of multiple infections, management of inflammatory responses and immune-mediated damage, as well as addressing gastrointestinal dysfunction and coagulation disorders. (Arefian et al., 2017; Cecconi et al., 2018; Marik, 2018).

## 3 Chinese medicine theory guides sepsis treatment

The fundamental principle of TCM is rooted in the “holistic concept,” which serves as a guiding principle, while the “syndrome differentiation” acts as the diagnostic and therapeutic method. These two theories direct the management of sepsis with TCM (Li and Xu, 2011). The “holistic concept” posits that the human body functions as an integrated organic entity, emphasizing the synergistic interplay among multiple organs to safeguard against disease and maintain homeostasis. The integration of “syndrome differentiation” and “disease differentiation” involves understanding the location, etiology, nature, and interplay between “positive” and “evil” factors, reflecting the pathological changes. The aforementioned statement also aligns with the concept of “personalized medicine” in contemporary medical practice, which holds immense significance in the realm of diagnosis and treatment. Western medicine research has shifted from a one-sided focus on eradicating disease-causing microorganisms to regulating the body’s immune response. It now emphasizes the intrinsic connection between organs in the whole body, as evidenced by theories such as liver-kidney syndrome, heart-kidney syndrome, and lung-intestinal axis. Traditional Chinese medicine is used to dynamically adjust multiple systems in patients, aligning with the holistic concept of Chinese medicine (Liu et al., 2022). Due to variations in individual characteristics, pathogenic factors, and disease duration, patients exhibit diverse responses to drug therapy. Additionally, each sepsis patient presents distinct clinical manifestations, disease progression levels, and alterations in disease mechanisms. Therefore, it is essential to employ diverse methods and treatment modalities tailored to the individual, timing, and location, aligning with the diagnostic and therapeutic approach of Chinese medicine known as “syndrome differentiation.”

The TCM treatment of sepsis can be summarized as “Four Syndromes and Four Methods” based on the etiology and pathogenesis. The “Four Syndromes” include blood stasis, toxic heat, viscera and qi obstruction as well as acute deficiency. Correspondingly, the “Four Methods” are activating blood circulation to remove stasis, clearing heat and detoxifying, regulating the lower energizer while nourishing the root (Xu and Lu, 2018). The improvement of microcirculation corresponds to the traditional Chinese medicine approach of “activating blood circulation to resolve stasis.” In sepsis, microcirculatory disorders are a significant pathological change characterized by inadequate capillary perfusion and sluggish blood flow. The objective of the “activating blood circulation to resolve stasis” in TCM is to enhance blood circulation, particularly microcirculation, thereby alleviating tissue hypoxia and ischemia, preventing the formation of microcirculatory thrombosis, as well as halting further progression of disseminated intravascular coagulation (DIC). The inhibition of inflammatory mediator release aligns with the treatment principle of “clearing heat and detoxifying.” A significant amount of inflammatory mediators such as tumor necrosis factor and interleukins are released during sepsis, resulting in systemic inflammation of the body. Traditional Chinese medicine’s approach to “clearing heat and detoxification” can mitigate inflammatory response through the utilization of specific TCM possessing heat-clearing and detoxifying properties, thereby suppressing excessive release of inflammatory mediators. Promoting the elimination of endotoxins is akin to the principle of “regulating the lower energizer” in traditional Chinese medicine. In sepsis, bacterial endotoxins accumulate within the body, exacerbating the condition. For septic patients presenting with symptoms such as constipation and abdominal distension, TCM that facilitate bowel movement and heat expulsion can be employed for sepsis treatment. Modern medicine acknowledges that the intestines serve as a major reservoir of bacteria in the human body. During severe infections like septicemia, stress weakens or damages intestinal barrier function, allowing a significant influx of bacteria and endotoxins into circulation via the portal vein and intestinal lymphatic system, resulting in gut-derived endotoxemia and bacterial translocation. The principle underlying both immune regulation and the “nourishing the root” method in traditional Chinese medicine is similar. Sepsis often leads to suppression or disruption of the body’s immune function. In traditional Chinese medicine, the emphasis on nourishing the spleen, kidneys, and other organs aims to enhance immune function, which can be seen as analogous to modern medical immunomodulatory treatments.

Combined with the clinical manifestations, the “method of supporting and consolidating the essence” should be considered as a fundamental treatment approach for “septic shock” or “sepsis recovery,” predominantly relying on TCM that promote and consolidate vital energy. “Activating blood circulation to remove stasis” is a treatment method used when shock is combined with DIC or to prevent the occurrence of DIC, primarily using traditional Chinese medicine that promotes blood circulation and resolves stagnation. “Clearing heat and detoxifying” and “regulating the lower energizer” are therapeutic approaches employed to combat pathogenic infections, while also serving as preventive measures against septic shock. These methods primarily focus on utilizing TCM with heat-removing properties to clear heat and toxins from the body’s lower internal organs (Li and Wang, 2017; Ling et al., 2023). The Chinese herbal medicine types that clear heat and detoxify, promote blood circulation, remove blood stasis, and strengthen the foundation of treatment are effective in treating sepsis. The “Four Syndromes and Four Methods” provide a convenient way to identify infectious shock in TCM. The four syndromes can simultaneously correspond to the various stages of pathology in Western medicine, thereby enhancing the precision in drug identification and utilization (Jing et al., 2022). The treatment of sepsis in TCM aims not only to eliminate pathogenic factors, but also to restore internal balance and coordination within the organism (Liu et al., 2022). It regulates the functions of internal organs, promoting harmony between the five viscera and six bowels, allowing the body to achieve an optimal state through medication for self-regulation. This ultimately restores internal homeostasis and realizes the delicate interplay of yin and yang.

## 4 Mechanism action of TCM extracts in treatment of sepsis

Traditional Chinese medicine extracts and its active components possess distinct chemical structures, precise therapeutic effects, and facilitate quality control. These compounds offer “individualized therapies” for different stages of sepsis development through multiple pathways and targets, thereby promoting body equilibrium (Han and Wu, 2010; Li et al., 2013). This review provides a comprehensive overview of the advancements in sepsis treatment research, focusing on the extraction and isolation of bioactive compounds from natural sources. These compounds are obtained through inhibiting the release of inflammatory mediators, facilitating endotoxin elimination, enhancing microcirculation, and modulating immune responses.

**FIGURE 1:**
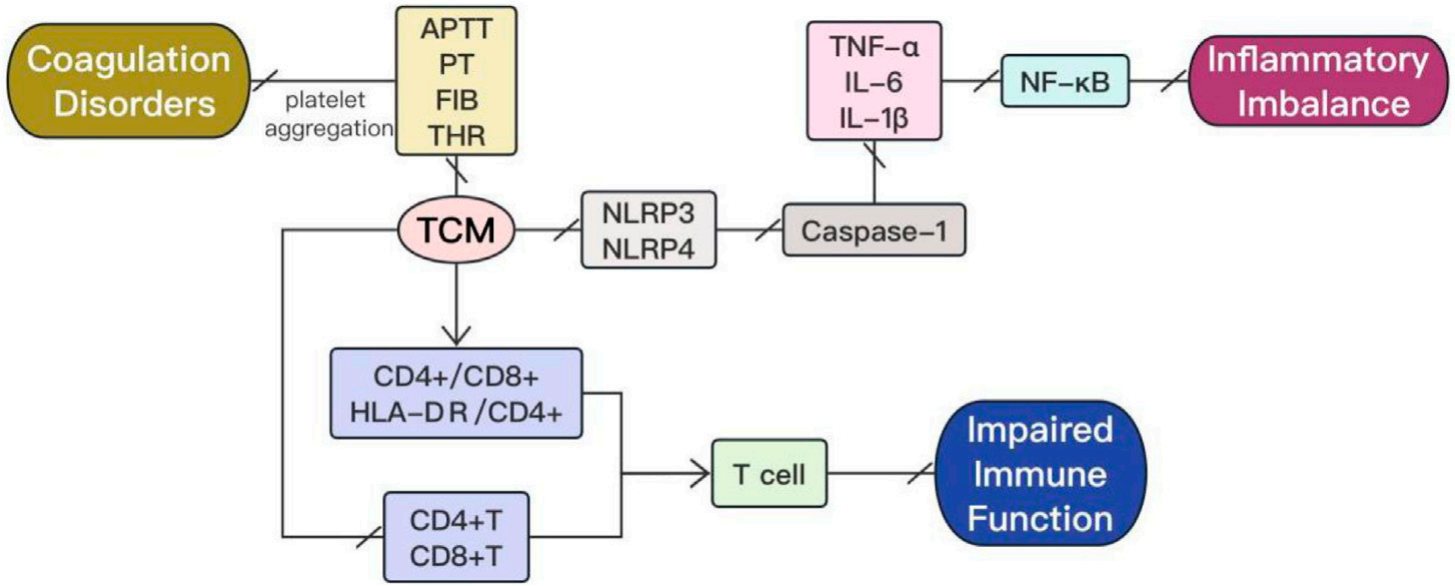
The mechanism actions in treatment of sepsis with TCM extracts involve the modulation of inflammatory imbalance, impaired immune function, and coagulation disorders.

**FIGURE 2:**
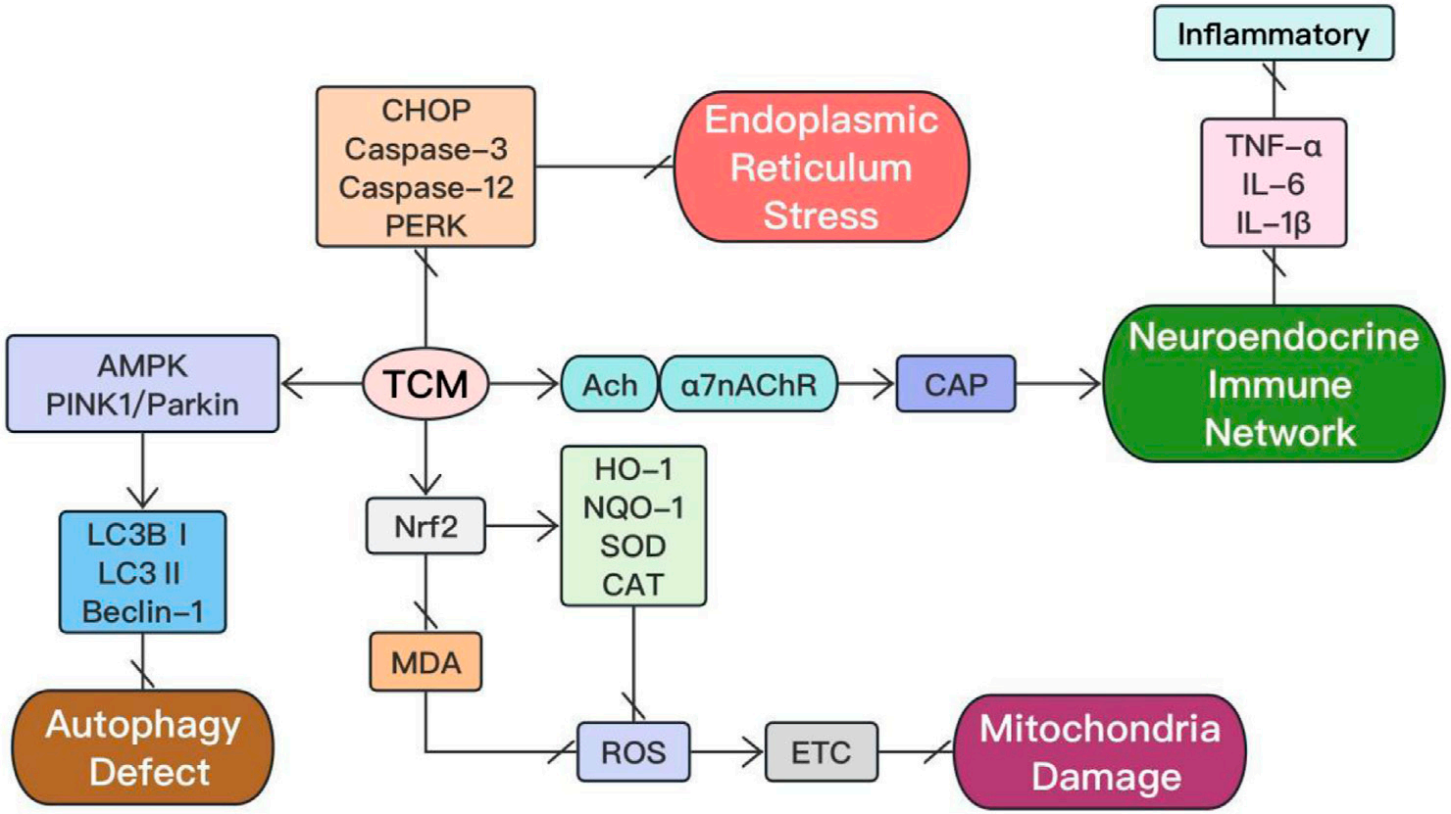
The other mechanism actions in treatment of sepsis with TCM extracts.

The mechanisms action of TCM extracts in the treatment of sepsis are systematically summarized and presented in Figures 1, 2. The extracts of traditional Chinese medicine regulate diverse mechanisms through various signaling pathways to enhance sepsis management, exemplifying the multifaceted approaches and targets characteristic of TCM in disease treatment.

### 4.1 Inflammatory imbalance

**FIGURE 3:**
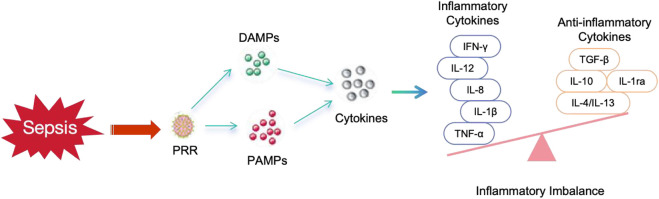
The inflammatory imbalance process during sepsis.

Inflammatory imbalance serves as the fundamental basis for the pathogenesis and progression of sepsis. The pathogenic microorganisms, including bacteria, fungi, parasites, and viruses, elicit responses throughout the course of sepsis. The initial acute response triggered by the host in response to an invasive pathogen typically involves phagocytosis of the pathogen by macrophages and production of a variety of pro-inflammatory cytokines. This leads to the onset of a cytokine storm and activation of the intrinsic immune system (Lin et al., 2022). The activation of the intrinsic immune system is clearly mediated by pattern-recognition receptor, which detect damage-associated molecular pattern (DAMP) or pathogen-associated molecular pattern (PAMP) and subsequently upregulate inflammation-related genes, initiating a cascade of immune cell activations (Raymond et al., 2017). In the immune response to sepsis, exogenous factors derived from pathogens, such as lipopolysaccharides, and endogenous factors released by injured cells both play a crucial role. Figure 3 shows the inflammatory imbalance process during sepsis. The findings of various studies have demonstrated the effective inhibition of inflammatory factor release, regulation of pro-inflammatory and anti-inflammatory responses, antagonism against endotoxins, inhibition of hyperfibrinolysis, as well as modulation of the expression of inflammatory signaling pathways by herbal monomers (Li et al., 2013). These mechanisms contribute to the protection of septic organs’ function and improvement in patient prognosis. The characteristics of TCM extracts by the modulation of inflammatory imbalance on sepsis are summarized in Table 1.

**TABLE 1:** Characteristics of TCM extracts by the modulation of inflammatory imbalance on sepsis.

Name	Source	Validation model	Administration	Effect	Organ	Positive control	Ref.
Salvianolic acid	*Salvia miltiorrhiza* Bunge	Children with sepsis and shock	200 mg/time	↓: CRP, PCT, NF-κB, HMGB1, sTREM-1	NA	NA	Xu (2022)
Salvianolic acid A	*Salvia miltiorrhiza* Bunge	LPS-induced C57 mice	125, 25, 5, and 1 μg/mL	↓: TNF-α、IL-6 ↑: IL-10	Lung	Dexamethasone (200 μg/mL)	Wu et al. (2020b)
Salvianolic acid A	*Salvia miltiorrhiza* Bunge	CLP-induced SD rats	100 mg/kg	↓: TNF-α, IL-1, IL-6, AST, ALT, ALP	Liver	NA	Wei et al. (2021)
Tanshinone IIA	*Salvia miltiorrhiza* Bunge	LPS-induced mice	10 mg/kg	↓: TNF-α, IL-1β	Brain	NA	Zhang et al. (2017)
Polydatin	*Polygonum cuspidatum* Siebold & Zucc.	CLP-induced SAKI mice	30 mg/kg	↓: MDA, TNF⁃α, IL⁃1β, IL⁃6 ↑: GSH/GSSG, SOD, T⁃AOC	Kidney	SIRT3 inhibitor 3-TYP (5 mg/kg)	Lei and Li (2021)
Resveratrol	*Veratrum album* L.	Patients undergoing TRUS biopsy CLP-induced male C57/BL6 mice	1 mg/kg body weight	↓: PLD, SphK1, ERK1/2, NF-κB, LPS/Toll-4/NF-κB, HMGB1, MyD88	NA	NA	Wang et al. (2020a)
*Astragalus* polysaccharides	*Astragalus membranaceus* (Fisch.) Bge. or A. membranaceus (Fisch.) Bge. var. *mongholicus* (Bge.) *Hsiao*	CLP-induced SD rats	100 mg/kg	↓: IL⁃6, TNF⁃α, ESM-1, TLR4/NF-κB ↑: HSP70	Lung	NA	Xu et al. (2023)
Isoliquiritigenin	*Glycyrrhiza uralensis* Fisch. ex DC.	LPS-induced male C57BL/6 mice	50 µM or 100 µM	↓: HMGB1 ↑: NCOA4	Kidney	Ferrostatin-1	Tang et al. (2021)
Murrayanine	*Murraya paniculata* (L.) Jack	LPS-induced RAW 264.7 cells and female Balb/c mice	10 mg/kg	↓: iNOS, COX -2, NO, PGE2, TNF-α, IL-6, NF-κB	Liver, Lung, Kidney	Dexamethasone	Gupta et al. (2019)
Ginsenoside Rg2 and Rh1	*Panax ginseng* C.A.Mey.	LPS-induced RAW 264.7 cells and ICR mice	20 mg/kg	↓: TLR4, NF-κB, TNF-α, IL-1β, IFN-β ↑: p38-STAT1	Liver, Kidney	Dexamethasone	Huynh et al. (2020)
Ginsenoside Rg1 and Re	*Panax ginseng* C.A.Mey.	LPS-induced RAW 264.7 and RAW-Blue cells LPS-induced male SD rats and BALB/c mice	30, 5, 70 μg/mL Rg1 and 50, 70 μg/mL Re 10 mg/kg Rg1 and 20 mg/kg Re	↓: TNF-α, IL-1β, IL-6, COX-2, iNOS, TLR4	NA	TAK-242 (1 mg/kg)	Su et al. (2015)
Phillyrin	*Forsythia suspensa* (Thunb.) Vahl	LPS-induced EA.hy926 cell and AKI mice	20 µM	↓: ROS, cathepsin L, heparinase, NF-κB/MAPK, IL-1β, IL-6, TNF-α	Kidney	Cathepsin L inhibitor	Zhang et al. (2020)
Puerarin	*Pueraria lobata* (Willd.) Ohwi	LPS-induced male BALB/C mice	50 mg/kg	↓: ICAM-1, VCAM-1, E-selectin, TNF-α, IL-1β	Lung	NA	Wang et al. (2019b)
Ethanol extract of *Cinnamomum cassia*	*Cinnamomum cassia* (L.) J.Presl	LPS-induced sepsis mouse model and MSU-induced gout model using male C57BL/6 mice	50, 100 mg/kg	↓: NLRP3, NLRC4, AIM2, IL-1, caspase-1	NA	zVAD	Shin et al. (2017)
Ligustrazine	*Ligusticum chuanxiong* S.H.Qiu, Y.Q.Zeng, K.Y.Pan, Y.C.Tang & J.M.Xu	CLP-induced male C57BL/6 mice Patients with congenital non-cyanotic heart disease undergoing open heart surgery	4 mg/kg, 40 mg/kg 3 mg/(kg·BW)	↓: MDA, MPO, IL-6, IL-8, TNF-α, ICAN-1	Lung Heart	NA	Lu et al., 2007; Zeng (2010)
*Astragalus* polysaccharides	*Astragalus membranaceus* (Fisch.) Bge. or *A. membranaceus* (Fisch.) Bge. var. *mongholicus* (Bge.) Hsiao	LPS-induced male C57BL/6 mice	100, 200, 400 mg/kg	↑: IL-2, IL-10, TGF-β ↓: IL-4, IL-17, TNF-α, Th1/Th2, Th17/Treg	Lung	NA	Xu and Liu (2022)
Curcumin	*Curcuma longa* L.	CLP-induced SD rats	50, 100, 200 mg/kg	↓: IL-6, IL-10, TNF-α	Lung	NA	Jiang et al. (2022)
Baicalin	*Scutellaria baicalensis* Georgi	LPS-induced male BALB/C mice	75, 150, 300 mg/kg	↓: W/D, PaCO_2_, Th17/Treg, TNF-α, IL-6, IL-17, MDA, TLR4/NF-κB ↑: TGF-β, SOD, OI, PaO_2,_ Treg/Th17	Lung	Dexamethasone (7.2 mg/kg)	Xu et al. (2022b)
Salidroside	*Rhodiola crenulate* (Hook.f. & Thomson) H.Ohba	CLP-induced male BALB/C mice	5, 10, 20 mg/kg	↓: α-SMA, FN, Vimentin, Col Ⅰ, p-JAK2, p-STAT3, iNOS, IL-6 ↑: IL-4, IL-10	Lung	NA	Guo et al. (2022)
Kukoamine B	*Lycium chinense* Mill.	LPS-induced KM mice	1.25, 2.5, 5 mg/kg	↓: TLR4, TLR9, iNOS, COX-2, NF-κB, TNF-α, IL-6	Lung, liver, kidney, heart	NA	Liu et al. (2011)
Berberine	*Coptis chinensis* Franch.	LPS-induced male KM mice LPS-induced male C57BL/6 mice	25, 50, 100 mg/kg 10 mg/kg	↓: TNF-α, IFN-γ, NO, NF-κB, IL-1β, IL-6 ↑: IL-10	Lung	NA	Li et al. (2006), Wang et al. (2020b)

The active components salvianolic acid and tanshinone, derived from the Chinese herbal medicine *Salvia miltiorrhiza* Bunge, possess biological effects such as enhancing circulation, exerting anti-inflammatory and antioxidant effects (Wang J. et al., 2019). These components can potentially serve as therapeutic agents against the release of inflammatory and oxidative mediators during sepsis and microcirculatory disorders in shock (Yuan J. et al., 2021). Xu (2022) discovered that the incorporation of salvianolic acid into conventional fluid resuscitation can effectively suppress the inflammatory and stress responses during sepsis combined with shock. Additionally, it has been shown to significantly enhance blood oxygen metabolism in pediatric patients with sepsis combined with shock. Wu S. et al. (2020) utilized lipopolysaccharide (LPS)-induced macrophage RAW264.7 and a murine sepsis model to assess the impact of metabolites isolated from extracts of the aerial parts of *S. miltiorrhiza*, specifically salvianolic acid A (administered via intraperitoneal injection at concentrations of 125, 25, 5, and 1 μg/mL), on sepsis. The cellular activity was evaluated using the methyl thiazolyl tetrazolium (MTT) assay, while the levels of inflammatory factors were measured in both cells and mouse livers through enzyme-linked immunosorbent assay (ELISA) analysis. The results demonstrated that salvianolic acid A increased cellular activity in all dosage groups. Moreover, the levels of inflammatory factors TNF-α and IL-6 were significantly or markedly reduced, while the level of IL-10 was significantly elevated in all dosage groups (except for the 5 μg/mL group). These findings suggest that salvianolic acid A effectively mitigated the impact of LPS on cellular activity. The effects of intraperitoneal administration of salvianolic acid A at a dosage of 100 mg/kg on liver injury in rats with cecal ligation and puncture (CLP) were analyzed and investigated (Wei et al., 2021). The expression levels of inflammatory markers TNF-α, IL-1, IL-6, as well as liver injury markers aspartate aminotransferase (AST), alanine aminotransferase (ALT), and alkaline phosphatase (ALP) were detected using ELISA. Additionally, liver tissues from the rats were subjected to pathological observation. The results demonstrated that compared to the sepsis control group, the salvianolic acid experimental group exhibited a significant reduction in levels of both liver injury markers and inflammatory markers. Zhang et al. (2017) investigated the interventional effects of tanshinone IIA (10 mg/kg) on cerebral microcirculation and neuroinflammatory responses in LPS-induced septic mice. Utilizing the BI2000 microcirculation image processing system, researchers observed that blood flow velocity of microvessels and microarteries in the soft meninges at different phases of each group’s mice through an open cranial window. Additionally, ELISA was employed to detect the levels of inflammatory factors in mouse brain hippocampal tissue. The results demonstrated a significant acceleration in both micro-artery and micro-vein blood flow velocity within the drug group compared to that of the model group, along with significantly reduced levels of TNF-α and IL-1β in cerebral hippocampus tissue when compared to those found in the model group. These findings indicated that tanshinone IIA could enhance cerebral microblood flow velocity, mitigate neuroinflammatory reactions, and exert protective effects on the brain.

Lei and Li (2021) reported that polydatin significantly reduced serum levels of inflammatory factors in SAKI mice. Wang B. et al. (2020) demonstrated that resveratrol exerts regulatory effects on downstream signaling molecules of Toll-like receptor 4 (TLR-4), sphingosine kinase 1 (SphK1), extracellular signal-regulated kinase 1/2 (ERK1/2), and NF-κB, leading to a reduction in cytokine production by human primary monocytes stimulated with LPS through phospholipase D (PLD). Moreover, it downregulates myeloid differentiation factor 88 (MyD88), which inhibits the LPS/Toll-4/NF-κB signaling pathway, thereby attenuating the production of cytokines, chemokines, and high mobility group protein B1 (HMGB1) by human primary monocytes stimulated with LPS and exerting anti-inflammatory effects.

Xu et al. (2023) discovered that *Astragalus* polysaccharides (APS), a kind of polysaccharides obtained from the roots of *A. membranaceus* (Fisch.) Bge. or *A. membranaceus* (Fisch.) Bge. var. *mongholicus* (Bge.) Hsiao, mitigated the acute inflammatory response in septic rats. Tang et al. (2021) observed that the utilization of isoliquiritigenin reduced the expression of HMGB1 and inhibited ferroptosis in a sepsis-induced model of AKI, thereby attenuating the inflammatory response.

The major compound isolated from *Murraya paniculata* (L.) Jack is murrayanine, which has been reported to possess significant antioxidant (Abu Bakar et al., 2007), anti-inflammatory (Gupta et al., 2010), and immunomodulatory activities (Shah et al., 2008). Gupta et al. (2019) discovered that murrayanine inhibited the expression of inducible nitric-oxide synthase (iNOS) and cyclooxygenase (COX-2), reduces the production of nitric-oxide (NO), prostaglandin E2 (PGE2), TNF-α, and IL-6, decreases NF-κB activity, ameliorates LPS-induced lung, liver and kidney injuries, as well as increases survival rates in mice with LPS-induced sepsis models. These findings demonstrated that murrayanine was an ideal natural compound and a new chemical entity for treating sepsis and other inflammatory diseases.

The anti-inflammatory effects of ginseng have been demonstrated in a variety of diseases (Kim et al., 2018; Xu H. et al., 2022). Ginseng contains a diverse range of bioactive compounds, and its therapeutic potential has been associated with neuromodulation, anticancer activity, lipid modulation, and antithrombotic activity (Ru et al., 2015). However, there is currently no definitive study comparing the relative efficacy of different components of ginseng in the treatment of sepsis. A study suggested that treatments with ginsenoside Rg2 and Rh1 might exhibit greater efficacy compared to single-component treatments (Huynh et al., 2020). Similarly, another study demonstrated that the combination of ginsenoside Rg1 and Re in the treatment of septic mice effectively reduced LPS-induced hyperthermia, leukocyte counts, and serum levels of pro-inflammatory mediators. Furthermore, this combination even exhibited a 90% increase in survival rate among lethally septic shocked mice. Subsequent analysis revealed that the effective anti-inflammatory effect could be attributed to the different distribution locations of these components (Rg1 being located both intracellularly and extracellularly while Re is located extracellularly), suggesting that combining various components of ginseng might yield superior therapeutic outcomes (Su et al., 2015).

Phillyrin exhibited inhibitory effects on reactive oxygen species (ROS) production, reduced the expression levels of histone L and heparinase both *in vitro* and *in vivo*, and suppressed the secretion of inflammatory cytokines to ameliorate renal function in AKI mice (Zhang et al., 2020). The isoflavone compound puerarin has demonstrated its ability to dilate coronary blood vessels, enhanced myocardial metabolism, exhibited antioxidant and anti-inflammatory activities, protect vascular endothelial function, and improve microcirculation (Wei et al., 2014). Puerarin pretreatment at a dose of 50 mg/kg for 4 days significantly attenuated the serum levels of intercellular adhesion molecule-1 (ICAM-1), vascular cell adhesion molecule-1 (VCAM-1), and E-selectin, as well as TNF-α and IL-1β in mice with LPS-induced endotoxemia (Wang S. et al., 2019). These findings suggested that puerarin effectively suppressed the release of inflammatory factors and adhesion molecules, thereby ameliorating LPS-induced vascular endothelial cell injury.

Modern studies have confirmed the therapeutic effect of *Cinnamomum cassia* (L.) J.Presl in sepsis by inhibiting the activation of inflammatory vesicles. Shin et al. (2017) utilized bone marrow-derived macrophage BMDMs to investigate the modulatory effect of 50 and 100 mg/kg *C. cassia* ethanol extract on inflammatory vesicle activation. They also established a mouse model of LPS-induced sepsis and a monosodium urate-induced gout model, observing that the compound increased the survival rate of mice with LPS-induced septic shock while inhibiting the activation of nucleotide-binding domain, leucine-rich repeat, and pyrin domain-containing protein 3 (NLRP3), NLRP4, and melanoma-deficiency factor 2 inflammatory vesicles through inhibition of apoptosis-associated granulocyte-like proteins oligomerization within CARD structural domains, consequently suppressing IL-1β and caspase-1 secretion.

A study replicating a mouse model of sepsis by CLP demonstrated that ligustrazine significantly attenuated the pulmonary inflammatory response during sepsis, leading to a significant reduction in plasma levels of IL-6 in ligustrazine-treated septic mice (Zeng, 2010). Clinical studies have also revealed that ligustrazine effectively decreased peripheral blood levels of IL-8 and TNF-α in patients undergoing extracorporeal circulation, potentially contributing to the management of systemic inflammatory response syndrome during extracorporeal circulation (Lu et al., 2007).

The relevant studies have demonstrated that *Astragalus* polysaccharide (APS) exhibits diverse pharmacological activities, including anti-inflammatory, antioxidant, anti-atherosclerotic, apoptosis inhibition, and ischemia-hypoxia protection (Zhou et al., 2018). Consequently, it is extensively employed in clinical settings as a crucial immunomodulator or antioxidant (Zhang et al., 2019). The sepsis mouse model was created using LPS and divided them into groups, with APS administered at different doses and saline interventions respectively (Xu and Liu, 2022). The results demonstrated a significant improvement in the imbalance of Th1/Th2 and Th17/Treg cells in septic mice treated with APS. Additionally, there was an increase in the levels of anti-inflammatory factors such as IL-2, IL-10, and TGF-β, while pro-inflammatory factors including IL-4, IL-17, and TNF-α were downregulated. Furthermore, a significant dose-dependent effect was observed.

Curcumin exhibited significant potential in reducing the levels of IL-6, IL-10, and TNF-α within the lung tissue of septic rats (Jiang et al., 2022). This suggested that curcumin effectively ameliorated inflammation levels, alleviated immune suppression, and mitigated damage to lung tissues in septic rats.

Baicalin (BA), a flavonoid compound found in the root of *Scutellaria baicalensis* Georgi, exhibits various pharmacological effects such as lipid-lowering, anti-inflammatory, antibacterial, antioxidant and immunomodulatory activities. The mice were induced with sepsis through intraperitoneal injection of LPS, followed by treatment with baicalin to mitigate lung injury (Xu L. et al., 2022). The results demonstrated that BA inhibited TLR4/NF-κB pathway activation, suppressed inflammatory response, promoted Treg/Th17 balance shift towards Treg cells, reduced lung tissue damage and improved lung function. The effect was dose-dependent.

Salidroside, the active component found in *Rhodiola crenulate* (Hook.f. & Thomson) H.Ohba, exhibits pharmacological effects that include anti-aging, anti-inflammatory, immunomodulatory, and antioxidant properties. A murine model of acute lung injury with sepsis was established through CLP (Guo et al., 2022). The findings demonstrated that salidroside attenuated the pulmonary response in CLP-induced septic mice by downregulating the expression of JAK2 and STAT3, while significantly reducing the levels of iNOS and IL-6 in lung tissue. Moreover, there was a significant increase in IL-4 and IL-10 levels, indicating that salidroside ameliorates lung injury in CLP-induced septic mice by modulating inflammatory factors within the lung tissue.

Kukoamine B is an alkaloid component primarily derived from the Chinese medicinal plant *Lycium chinense* Mill. In a study, the affinity of kukoamine B towards LPS and CpG DNA was investigated, along with its inhibitory effect on the release of TNF-α and IL-6 from RAW264.7 cells both independently and in combination (Liu et al., 2011). The findings demonstrated that kukoamine B exhibited significant neutralization of multiple pathogenic molecules and effectively suppressed the induced inflammatory response. Notably, kukoamine B represents the first reported natural product-derived inhibitor targeting both LPS and CpG DNA.

Berberine, a bioactive alkaloid isolated from Chinese herbal medicine *Coptis chinensis* Franch., has been shown to reduce plasma levels of TNF-α, IFN-γ and NO in mice with sepsis (Li et al., 2006). Recent domestic research also confirmed that berberine could inhibit the activation of NF-κB signal transduction and further supports its use as a single drug for treating sepsis patients along with other drugs (Wang Y. et al., 2020).

In conclusion, TCM extracts with anti-inflammatory properties effectively suppress the release of inflammatory factors, regulate pro-inflammatory/anti-inflammatory balance, and modulate the expression of inflammatory signaling pathways in organs affected by sepsis (show Figure 1). Therefore, these TCM extracts hold tremendous potential for treating sepsis.

### 4.2 Impaired immune function

The dysfunction of immune function is a crucial factor contributing to the occurrence and progression of sepsis. During the inflammatory response in sepsis, neutrophils interact with and migrate across endothelial cells under the influence of chemokines that are attracted to the site of inflammation. At this location, they recognize and engulf pathogens, release various active factors and proteolytic enzymes, ultimately eliminating the pathogen (Shen et al., 2017). Severe impairment of the immune system is a common pathological change observed in sepsis. During the initial cytokine storm phase, a significant number of sepsis patients may succumb to death. While survivors may experience immune suppression, leading to an inability to clear primary infections and reactivation of latent viruses. Sepsis-induced immunosuppression affects both innate and adaptive immunity. The immunosuppressive response following sepsis has been characterized as a compensatory anti-inflammatory response syndrome, which is regulated by co-stimulatory molecules like CD80/B7-1 that are produced through activation of the TLR signaling pathway. Additionally, this response involves the conversion of naive T cells into cytokine-induced regulatory T cells, resulting in decreased expression of antigen-presenting associated transcription factors (e.g., CD80/B7-1) and IRF4/MUM1 (Honma et al., 2005; Negishi et al., 2005; Pepin et al., 2018). Traditional Chinese medicine has the ability to regulate the functions of immune cells, including their composition, differentiation, activation, secretion, and killing (Yang and Fan, 2021). This enables it to enhance the body’s innate resistance against pathogens and prevent cytokine storms. Specifically, it can interrupt and reverse the immunosuppressive state associated with severe sepsis, thereby improving prognosis and preventing further damage caused by pathogenic toxins (Wang and Chen, 2017). The characteristics of TCM extracts by the modulation of impaired immune function on sepsis are summarized in Table 2.

**TABLE 2:** Characteristics of TCM extracts by the modulation of impaired immune function on sepsis.

Name	Source	Validation model	Administration	Effect	Organ	Positive control	Ref.
*Astragalus* injection	*Astragalus membranaceus* (Fisch.) Bge. or *A. membranaceus* (Fisch.) Bge. var. *mongholicus* (Bge.) Hsiao	Patients with traumatic splenic rupture after splenectomy	20 mL in 250 mL 5% glucose	↑: CD3^+^, CD4^+^, CD4 +/CD8+ ↓: CD8^+^	NA	NA	Gao et al. (2018)
*Astragalus* granule	*Astragalus membranaceus* (Fisch.) Bge. or *A. membranaceus* (Fisch.) Bge. var. *mongholicus* (Bge.) Hsiao	Patients with sepsis	1.0, 2.0, 3.0 g/time/d	↑: CD4^+^, CD4 +/CD8+ ↓: CD8^+^, NK cell	NA	NA	Xu et al. (2022c)
*Rhubarb* preparation	*Rheum palmatum* L.	Patients with sepsis	50 g in 50 mL Physiological saline (2 times/d)	↓: TNF-α, IL-6, IL-1β ↑: IL-10, CD4+/CD8+, HLA-DR/CD4+	NA	NA	Yin and Guo (2015)
Artesunate	*Artemisia annua* L.	CLP-induced male BALB/c mice	10 mg/kg	↓: CD4+/CD8+, PD-1, CTLA-4, BTLA, caspase-9 ↑: TNF-α, CD4+T, CD8+T, MAPK/ERK	Lung, spleen	NA	Yuan et al. (2023)
Ginsenoside Rg1	*Panax ginseng* C.A.Mey.	CLP-induced male 57BL/6 mice	20 mg/kg	↓: TNF-α, IL-6, IL-10, NO ↑: neutrophil	Spleen, thymus, liver, lung	NA	Zou et al. (2013)
Salidroside	*Rhodiola crenulate* (Hook.f. & Thomson) H.Ohba	CLP-induced male C57BL mice	20 mg/kg	↓: IL-6, IL-2, IL-10, CXCL-10, CD4+T, CD8+T	Thymus	NA	Pan et al. (2022)
Baicalin	*Scutellaria baicalensis* Georgi	Endotoxin-induced SD rats	100, 200 mg/kg/d	↓: TNF-α, IL-6, caspase-1, caspase-11 ↑: CD3+T/CD4+T, CD4+/CD8+, Th1/Th2	Lung	Ulinastatin (200,000 U/kg)	Yang et al. (2022)

The advanced study have demonstrated that *Astragalus* injection exhibits a significant improvement effect on T lymphocyte subsets in patients with traumatic splenectomy, while maintaining high levels of drug safety (Gao et al., 2018). This suggested that *Astragalus* injection possessed the ability to modulate various immune active cells, thereby ameliorating immune imbalance through metabolic regulation and exerting dual regulatory effects on immune function. The immunomodulatory effects of high-dose Huangqi granules was further validated by promoting lymphocyte proliferation and regulating the proportion of lymphocyte subsets through rigorous investigations (Xu X. et al., 2022).

Yin and Guo (2015) utilized *Rhubarb* preparation for small intestine perfusion via naso-intestinal tube and observed a significant reduction in the levels of TNF-α, IL-6, and IL-1β among sepsis patients. Simultaneously, there was an increase in the levels of IL-10, as well as improvements in CD4+/CD8+ ratios and human leukocyte antigen-DR (HLA-DR)/CD4+ expression. These findings provided evidence supporting the immunomodulatory effects of *Rhubarb* preparation on sepsis patients.

An immunosuppressive mouse model of CL-induced sepsis-related secondary bacterial infection was established, demonstrating that artesunate could reverse immunosuppression not only through innate immune regulation but also through adaptive immune regulation (Yuan et al., 2023). These immunosuppressive effects on the adaptive immune response were achieved by increasing the number of T cells through inhibition of CD4^+^ and CD8^+^ T cell apoptosis. Artesunate inhibited the expression of programmed cell death protein-1 (PD-1), cytotoxic T lymphocyte antigen-4 (CTLA-4), and B and T lymphocyte attenuator (BTLA) receptors while activating the MAPK/ERK signaling pathway as its potential mechanism. Furthermore, it was found that artesunate can inhibit caspase-9 expression and activation, potentially specifically targeting the mitochondrial apoptosis pathway in T cells. These findings suggested that artesunate had the potential to function as a modulator for both T cell number and function, making it a promising candidate for treating immunosuppressive diseases.

The intravenous administration of 20 mg/kg ginsenoside Rg1 to CLP mice resulted in an increase in peritoneal neutrophil counts (Zou et al., 2013). Additionally, the application of ginsenoside demonstrated inhibitory effects on thymus and spleen lymphocyte apoptosis, thereby enhancing bacterial clearance rate and survival rate. These findings suggested that ginsenoside Rg1 had the potential to enhance innate immunity, promote maintenance of adaptive immunity, and provide effective protection against sepsis.

Salidroside is derived from the dried roots and rhizome of *R. crenulate* (Hook.f. & Thomson) H.Ohba*,* possessing anti-cancer, antioxidant, anti-inflammatory, and immunomodulatory properties (Magani et al., 2020). Researches indicated its significant role in improving neuroinflammation (Wang et al., 2018), inhibiting renal fibrosis (Li et al., 2019), ameliorating fatty liver conditions (Zheng et al., 2018), and suppressing cancer cell proliferation (Zhu et al., 2020). Pan et al. (2022) established a sepsis mouse model induced by CLP and divided the mice into groups. They were treated with either salidroside or saline solution. The administration of salidroside significantly reduced the expression of inflammatory factors TNF-α, IL-6, IFN-γ, as well as inhibitory factor IL-10 in the spleen of septic mice. This suggested that salidroside could inhibit the inflammatory response in the spleen of septic mice and subsequently regulate their splenic immune function. Additionally, salidroside was found to inhibit CXC chemokine ligand-10 (CXCL-10) expression in the spleen of mice with sepsis, thereby reducing infiltration by immune cells and improving splenic immune status.

After baicalin intervention, there were a decrease in the elevated levels of TNF-α and IL-6 in the serum of septic rats, as well as an increase in the percentages of CD3^+^ T lymphocytes, CD4^+^ T lymphocytes, CD4+/CD8+ ratio, and Th1/Th2 ratio in peripheral blood (Yang et al., 2022). No significant difference between the high-dose baicalin group and ulinastatin group was observed when compared to each other. These findings suggested that baicalin had the potential to downregulate inflammatory factors and improve immune disorders in septic rats.

Traditional Chinese medicine extracts can regulate the body’s immune function and improve immune disorders (show Figure 1). They play a crucial role in reversing the immunosuppressive state of severe sepsis and enhancing the body’s own resistance.

### 4.3 Coagulation disorders

The current research has identified the interaction between inflammation and coagulation as a pivotal mechanism in the pathogenesis of sepsis. The inflammatory response in sepsis triggers a coagulation reaction and initiates coagulation, while the ensuing coagulation response further amplifies the inflammatory process (Ma et al., 2019). Under normal circumstances, three key physiological factors regulate the activation of coagulation through anticoagulant pathway systems: the tissue factor pathway inhibitor system, the activated protein C (APC) system, and the antithrombotic system that controls coagulation activation (Petäjä, 2011). The three pathways in sepsis exhibit varying degrees of disruption. Owing to impaired protein synthesis, the coagulation inhibitor pathways demonstrate sustained depletion and protein degradation at low levels. The downregulation of thrombomodulin (TM) and endothelial protein C receptor expression is attributed to the conversion of protein C to APC under inflammatory conditions (Levi and Poll, 2015). The maximal activation of endogenous fibrinolysis and coagulation is significantly attenuated in sepsis, wherein the production of fibrinogen activators (i.e., tissue plasminogen activator (t-PA)) and urokinase-type plasminogen activator (u-PA) by vascular endothelial cell storage sites leads to an augmented stimulation of fibrinogen activation and release of subquantified plasmin, while this effect is counteracted by the sustained increase inhibitor-1 (PAI-1) (Biemond et al., 1995). The Chinese medicines extracts can exert their effects on various components including blood vessels, endothelial cells, platelets, and coagulation factors. They possess the ability to inhibit platelet aggregation and activation, thereby improving sepsis-associated coagulation disorders and reducing the incidence of DIC, ultimately enhancing the quality of life for sepsis patients (Jin et al., 2022). The characteristics of TCM extracts by the modulation of coagulation disorders on sepsis are summarized in Table 3.

**TABLE 3:** Characteristics of TCM extracts by the modulation of coagulation disorders on sepsis.

Name	Source	Validation model	Administration	Effect	Organ	Positive control	Ref.
*Persicae semen* extract	*Prunus persica* (L.) Batsch	SD rats model of stasis-heat syndrome	2, 4, 8 g/kg	↓: CD31 ↑: NF-κB, IκB	Kidney	*Salvia miltiorrhiza* (14 g/kg)	Yi et al. (2016)
Huangqi (*Astragalus*) and Danggui (*Angelica*)	*Astragalus membranaceus* (Fisch.) Bge. or *A. membranaceus* (Fisch.) Bge. var. *mongholicus* (Bge.) Hsiao *Angelica sinensis* (Oliv.) Diels	Male ICR mice with lung fibroblasts model	0.1 mL/10 g (the dosage of Huangqi and Danggui is 50 g:10 g, 30 g:30 g, 10 g:50 g, 25 g: 5 g, 15 g:15 g, 5 g:25 g)	↓: Hyp	Lung	NA	Li et al. (2015)
Breviscapine	*Erigeron breviscapus* (Vaniot) Hand.-Mazz.	SD rats with OGD model	10, 30, 90 mg/L	↓: LDH, TNF-α, IL-6, IL-1β, PAI-1, ET-1 ↑: t-PA, PGI_2_	Brain	NA	Li et al. (2017b)
Safflower yellow A	*Carthamus tinctorius* L.	CLP-induced male SD rats	20 mg/kg	↓: PT, APTT, TFPI ↑: IL1-β, TNF-α, TF	NA	NA	Li and Jin (2016)
Emodin	*Rheum palmatum* L.	Male Tuck-Ordinary mice with cerebrovascular thrombosis model	4 mg/kg	↓: TNF-α, IL-1β ↑: SOD, PT, aPTT	Heart	NA	Nemmar et al. (2015)
Emodin-6-*O*-b-D-glucoside	*Rheum palmatum* L.	CLP-induced male C57BL/6 mice	0.1, 0.2, 0.5, 1, 2, 5, 10 μM	↓: TACE, EPCR, p38, ERK1/2, JNK	NA	NA	Lee et al. (2013)
Emodin	*Rheum palmatum* L.	CLP-induced male SD rats	50 mg/(kg·d)	↓: APTT, MA, CI, FLEV	NA	NA	Lin et al. (2018)
Baicalin	*Scutellaria baicalensis* Georgi	Endotoxin-induced SD rats	100, 200 mg/kg/d	↓: APTT, PT, FIB	Lung	Ulinastatin (200,000 U/kg)	Yang et al. (2022)

*Persicae semen* extract possessed inhibitory effects on platelet aggregation, the release of various inflammatory cytokines, inflammation-induced vascular permeability, and pulmonary vascular endothelial damage reduction (Yi et al., 2016). Huangqi (*Astragalus*) and Danggui (*Angelica*) were revealed the ability to induce relaxation in airway smooth muscle, scavenge free radicals, inhibit platelet aggregation, and suppress the release of inflammatory mediators such as thromboxane (Li et al., 2015). These actions effectively counteract microthrombosis and confer a protective effect on the vascular endothelium. In another study, breviscapine effectively enhanced the regulation of cellular coagulation and fibrinolysis (Li Z. et al., 2017).

A sepsis model was established by inducing CLP in SD rats (Li and Jin, 2016). Simultaneously, safflower yellow A was administered at various doses, revealing that a low dose of safflower yellow A effectively attenuated the prolongation of prothrombin time (PT) and activated partial thromboplastin time (APTT). Furthermore, it significantly suppressed the expression levels of serum TF, IL-1β, and TNF-α, while enhancing the expression level of serum tissue factor pathway inhibitor (TFPI). These findings suggested that a low dose of safflower yellow A could alleviate the reciprocal amplification between coagulation and inflammation during sepsis, ameliorate sepsis-induced coagulation dysfunction, regulate inflammatory factors’ expression, delay organ dysfunction syndrome onset, and exert protective effects against lung injury in septic conditions.

The therapeutic effects of emodin on cardiovascular responses induced by diesel exhaust particles (DEP) were investigated in a mouse model (Nemmar et al., 2015). They discovered that emodin significantly reduced the levels of inflammatory factors such as leukocytes, TNF-α, and IL-1. Moreover, it effectively inhibited platelet aggregation *in vitro*, prevented DEP-induced shortening of APTT and PT, while improved the pre-thrombotic state of small cerebral arteries and veins. The intravenous administration of emodin-6-*O*-b-D-glucoside in CLP model mice effectively attenuated the release of endothelial cell protein c receptor (EPCR) induced by CLP through inhibition of TNF-α converting enzyme (TACE) expression, thereby exerting a modulatory effect on anticoagulation (Lee et al., 2013).

CLP-introduced rats with sepsis exhibited a hypocoagulable tendency attributed to the attenuation of coagulation factors and fibrinogen function (Lin et al., 2018). Moreover, it was observed that the endogenous coagulation factor function and fibrinogen function of CLP rats improved, leading to a reduction in 24-h mortality rate following intervention with emodin (50 mg/kg.d). After baicalin intervention, a significant reduction in the levels of APTT, PT, and fibrinogen (FIB) in the sepsis group of rats was observed, particularly in the high dose group of baicalin (Yang et al., 2022). These findings suggested that baicalin effectively ameliorated coagulation disorders in septic rats and disrupted the coagulation-inflammation vicious cycle that mutually exacerbated each other.

Therefore, TCM extracts hold back platelet aggregation and activation, improve coagulation dysfunction in sepsis, as well as reduce the incidence of disseminated intravascular coagulation and multiple organ dysfunction syndrome, enhancing the prognosis of septic patients (show in Figure 1).

### 4.4 Other mechanisms

#### 4.4.1 Neuroendocrine immune network

The homeostatic regulation involved in the interaction between the neuroendocrine and immune systems constitutes a crucial component of the host response during septic shock (Muscatell et al., 2015). The central nervous system’s response to sepsis relies on three primary mechanisms: (1) circulatory inflammatory mediators that communicate with the central nervous system through the choroid plexus and ventricular organs; (2) involvement of the autonomic nervous system, where primary afferent nerves and sensory nerves interact with PAMPs, thereby amplifying inflammatory cytokine activation; (3) activation of endothelial cells in the blood-brain barrier, leading to the release of inflammatory mediators like NOS metabolites (Sonneville et al., 2013). Additionally, dysfunction of the hypothalamic-pituitary-adrenal axis results in reduced serum levels of adrenocorticotropin, adrenocorticotropin, and adrenocortisol in sepsis patients, leading to adrenal insufficiency syndrome (Kanczkowski et al., 2015). The evidence suggests that norepinephrine responds to LPS by exerting inhibitory effects on the expression of pro-inflammatory factors, such as TNF-β and IL-12, while simultaneously promoting the expression of anti-inflammatory cytokines like IL-10 (Tynan et al., 2012). There is limited research on monomer treatment of neuroendocrine immune network; however, recent studies have proposed the concept of the cholinergic anti-inflammatory pathway (CAP) in elucidating the involvement of the vagus nerve in sepsis regulation. The CAP represents a crucial neuro-immune regulatory pathway that activates α7 nicotinic ACh receptors (α7nAChR) through vagal stimulation, leading to acetylcholine (ACh) release and subsequent inhibition of pro-inflammatory cytokine synthesis and release. Consequently, targeting this inflammatory response offers a promising therapeutic approach for sepsis (Fujii et al., 2017). The field of traditional Chinese medicine encompasses a diverse range of plant species, with its active constituents primarily consisting of polysaccharides, glycosides, and alkaloids. It is plausible that certain components within traditional Chinese medicine exert their effects through cholinergic receptor modulation. Its anti-inflammatory and immunomodulatory properties are associated with the inhibition of inflammatory cytokine production, while also exhibiting bidirectional regulatory effects on cytokine synthesis and secretion by inflammatory cells. Thus, it exerts its actions via CAP (Deng et al., 2017). The characteristics of TCM extracts by the modulation of neuroendocrine immune network on sepsis are summarized in Table 4.

**TABLE 4:** Characteristics of TCM extracts by the modulation of neuroendocrine immune network, endoplasmic reticulum stress, autophagy defect, and mitochondria damage on sepsis.

Name	Source	Validation model	Administration	Target mechanism	Effect	Organ	Positive control	Ref.
*Scutellaria baicalensis* extract	*Scutellaria baicalensis* Georgi	LPS-introduced male SD rats	1.5, 3.0 g/kg	Neuroendocrine immune network	↓: NO, TNF-α, IL-1β, IL-6, IL-18 ↑: ACh	Lung	Galantamine (5 mg/kg)	Cui et al. (2012)
Puerarin	*Pueraria montana* var. *lobata* (Willd.) Maesen & S.M.Almeida ex Sanjappa & Predeep	D-galactose model ICR mice	60, 120 mg/kg	Neuroendocrine immune network	↑: ChAT, ACh	Brain	NA	Pan et al. (2008)
Paeoniflorin	*Paeonia lactiflora* Pall.	MCAO model SD rats	2.5, 5, 10 mg/kg	Neuroendocrine immune network	↓: M2 receptor ↑: M1 group receptor, ACh, Kir6.2/Kir6.1	Brain	NA	Wang et al. (2012)
Paeoniflorin	*Paeonia lactiflora* Pall.	Human umbilical vein endothelial cells after ischemia reperfusion	0.078, 0.156, 0.312 mg/mL	Neuroendocrine immune network	↓: ICAM-1, VCAM-1, p-p38 MAPK	NA	NA	Wang et al. (2016)
2′-Hydroxycinnamaldehyde (HCA), 2’- Benzoyloxycinnamaldehyde (BCA)	*Cinnamomum cassia* (L.) J.Presl	LPS-stimulated microglial cultures and microglia/neuroblastoma cocultures	HCA (2 μM), BCA (1 μM)	Neuroendocrine immune network	↓: ERK1/2, JNK, p38 MAPK, NF-κB, iNOS, IL-1β, TNF-α, NO	NA	NA	Hwang et al. (2011)
Genipin	*Gardenia jasminoides* J.Ellis	CLP-introduced male C57BL/6J mice	2.5 mg/kg	Endoplasmic reticulum stress	↓: CHOP, caspase-3, GRP78, p-eIF2α, PERK	Spleen	NA	Luo et al. (2023)
Resveratrol	*Veratrum album* L.	LPS-introduced male New Zealand white rabbits	250 mg/kg	Endoplasmic reticulum stress	↓: PERK, ATF4, CHOP, caspase9, Bax ↑: Bcl-2	Lung	NA	Han et al. (2020)
Ginsenoside Rb1	*Panax ginseng* C.A.Mey.	CLP-introduced female C57BL/6 mice	20 mg/kg	Endoplasmic reticulum stress	↓: GRP78, caspase-12	Heart, lung	NA	Huang et al. (2019b)
Allicin	*Allium sativum* L.	CLP-introduced male Balb/c mice	30 mg/kg	Autophagy defect	↑: SOD, LC3B, Beclin-1 ↓: TNF-α, IL-6, MDA	Lung	3-MA (15 mg/kg)	Peng et al. (2017)
Allicin	*Allium sativum* L.	CLP-introduced male Balb/c mice	30 mg/kg	Autophagy defect	↑: SOD, LC3II/I, Beclin-1 ↓: GPT, GOT, TNF-α, IL-6, MDA	Liver	NA	Gao et al. (2019)
Artesunate	*Artemisia annua* L.	LPS-introduced wide type male BALB/c mice	20 μg/mL	Autophagy defect	↑: LC3II/I, LC3B, NF-κB p65, p-Ser317/p-Ser637 ↓: TNF-α, IL-6, AMPK	Spleen	3-MA, ULK-101, SKF-96365, Bafilomycin A1	Liu et al. (2020)
Genipin	*Gardenia jasminoides* J.Ellis	CLP-introduced male imprinting control region mice	1, 2.5, 5 mg/kg	Autophagy defect	↓: calpain 1 ↑: Atg12-Atg5, Atg3, Rab7	Liver	NA	Cho et al. (2016)
Ginsenoside Rg3	*Panax ginseng* C.A.Mey.	CLP-introduced male C57BL/6 mice	6.25, 12.5, 25 μM	Autophagy defect	↑: OCR, MTP, AMPK, Beclin-1 ↓: ROS, AMPK	Liver	NA	Xing et al. (2017)
Ginsenoside Rg1	*Panax ginseng* C.A.Mey.	LPS-introduced C57BL/6 mice	40 mg/kg	Autophagy defect	↓: cleaved caspase 3, cleaved caspase-8 ↑: LC3II/LC3I	Lung	NA	Ji et al. (2019)
Ginsenoside Rg1	*Panax ginseng* C.A.Mey.	CLP-introduced male C57BL/6 mice	40, 200 mg/kg	Autophagy defect	↓: Iba1, TNF-α, IL-6, IL-1β, cleaved caspase 3, LC3-II, p62	Brain	NA	Li et al. (2017a)
*Radix hedysari* alcohol extract	*Hedysarum polybotrys* Hand.-Mazz.	CLP-introduced male SD rats	1.0, 2.0, 4.0 g/kg/d	Mitochondria damage	↑: HDL, IL-10, pNrf2/Nrf2, HO-1, NQO-1 ↓: AST, ALT, TC, TG, IL-6, MCP-1, TNF-α	Liver	NA	Yuan et al. (2021a)
Resveratrol	*Veratrum album* L.	CLP-introduced male Wistar albino rats	100 mg/kg	Mitochondria damage	↓: MDA, TNF-α ↑: GSH, SOD, GPX	Kidney, liver	NA	Aydın et al. (2016)
Resveratrol	*Veratrum album* L.	LPS-introduced male ICR mice	0.3 mg/kg	Mitochondria damage	↓: iNOS, NO, MDA, H_2_O_2_ ↑: GSH/GSSG, T-AOC, SOD, CAT	Lung	NA	Zhang et al. (2014)
Polydatin	*Polygonum cuspidatum* Siebold & Zucc.	LPS-introduced wild-type C57 mice	30 mg/kg	Mitochondria damage	↓: ROS ↑: SIRT3, SOD2, CypD	Lung, kidney, liver	NA	Wu et al. (2020a)
Polydatin	*Polygonum cuspidatum* Siebold & Zucc.	CLP-introduced C57BL/6 mice	30 mg/kg	Mitochondria damage	↓: ROS ↑: CLS1, CL, ATP, MMP	Kidney	3⁃TYP (5 mg/kg)	Lei et al. (2022)
*Astragalus* polysaccharides	*Astragalus membranaceus* (Fisch.) Bge. or *A. membranaceus* (Fisch.) Bge. var. *mongholicus* (Bge.) Hsiao	CLP-introduced SD rats	100, 200 mg/kg	Mitochondria damage	↓: ROS ↑: Na^+^ -K^+^ -ATP, SIRT1, PGC-1α, p-AMPK/AMPK	Kidney	Dexamethasone (10 mg/kg)	Wang et al. (2021b)
Ligustrazine	*Ligusticum chuanxiong* S.H.Qiu, Y.Q.Zeng, K.Y.Pan, Y.C.Tang & J.M.Xu	Patients with severe sepsis with myocardial injury	80 mg + GS/NS 250 mL	Mitochondria damage	↓: cTnT, MDA, ROS ↑: SOD	Heart	NA	Chen (2011)
Danhong injection	*Salvia miltiorrhiza* Bunge and *Carthamus tinctorius* L.	CLP-introduced male Wistar rats	5 mL/kg	Mitochondria damage	↓: MDA, HMGB1 ↑: SOD	Heart	NA	Hu and Lou (2016)

The administration of *S. baicalensis* extract in rats with acute lung injury resulted in an increase in serum ACh levels and a decrease in serum TNF-α and NO levels, effectively inhibiting the progression of inflammatory response (Cui et al., 2012). However, there were no significant effects observed on choline acetyltransferase (ChAT) and acetylcholinesterase (AChE) activities related to ACh synthesis and decomposition. Therefore, it can be inferred that the cholinergic anti-inflammatory pathway modulated by *S. baicalensis* extract may be attributed to its ability to enhance ACh release, which subsequently binds to nicotinoid α7 receptors or M receptors on monocytes, leading to a significant inhibition of TNF-α through intracellular signal transduction pathways. This mechanism also suppresses the release of proinflammatory factors IL-1β, IL-6, and IL-18, thereby regulating the overall inflammatory response.

Puerarin was found to enhance choline acetyltransferase activity and increase ACh content in the cerebral cortex of D-galactose rats (Pan et al., 2008). However, it did not exert a significant effect on cholinesterase activity. These findings suggest that puerarin’s anti-inflammatory effect may be attributed to its ability to activate the vagus nerve. Experimental studies have demonstrated that paeoniflorin exerts a protective effect on inflammatory cells through the regulation of cholinergic M receptor signaling pathway, modulation of cholinergic M receptor activity, and activation of the M-receptor-G protein-KATP channel (Wang et al., 2012). Paeoniflorin was postulated to exert its inhibitory effects on adhesion factors and the p38MAPK signaling pathway through the regulation of the cholinergic anti-inflammatory pathway, as demonstrated in experimental models (Wang et al., 2016). This regulatory mechanism ultimately leads to an amelioration of inflammatory responses in endothelial cells, alleviation of tissue damage, and a protective role in HUVECs.

2′-hydroxycinnamaldehyde (HCA) and 2′-benzoyloxycinnamaldehyde (BCA) in LPS-stimulated microglia cultures and microglia/neuroblastoma cocultures were observed to have the potential anti-neuroinflammatory effects, which protected neuroblastoma cells from microglia-mediated cell death (Hwang et al., 2011). It is postulated that low density lipoprotein receptor-related protein 1 (LRP1) serves as a promising molecular target for HCA in modulating microglial responses. The findings demonstrated that treatment with HCA at a concentration of 2 μmol/L and BCA at a concentration of 1 μmol/L effectively suppressed the expression of iNOS, proinflammatory cytokines IL-1β and TNF-α, as well as NO production by inhibiting the activation of ERK1/2, JNK, p38 MAPK, and NF-κB signaling pathways. These results highlight the therapeutic potential of HCA and BCA in managing neuroinflammatory diseases.

In summary, TCM extracts may inhibit the release of pro-inflammatory cytokines through the cholinergic anti-inflammatory pathway, playing an anti-neuroinflammatory effect and ultimately reducing tissue damage in sepsis (show in Figure 2).

#### 4.4.2 Endoplasmic reticulum stress

The endoplasmic reticulum is an intracellular organelle involved in the translocation, folding, post-translational modifications, and subsequent transport of intracellular organelles to the Golgi apparatus (Khan et al., 2015). In sepsis, accumulation of unfolded or misfolded proteins in the endoplasmic reticulum disrupts its homeostasis and leads to oxidative stress and severe calcium disturbances, resulting in endoplasmic reticulum stress (Khan et al., 2015). During endoplasmic reticulum stress, the unfolded protein response sensor may undergo a unique signal switch to induce cell death. The registration signaling mechanism involves several sequential steps: Firstly, the transcriptional activation of the C/EBP homologous protein (CHOP) gene is mediated by pkr-like endoplasmic reticulum kinase (PERK), inositol-requiring enzyme 1 (IRE1), and transcriptional activator 6 (ATF6). Secondly, there is an activation of the IRE-mediated JNK pathway, followed by TNF receptor-associated activator 2 and apoptosis signal-regulated kinase 1. Finally, activated caspase-12 associated with caspase-12 activation migrates from the endoplasmic reticulum to the cytoplasm, leading to cleavage of caspase-9 and ultimately activating caspase-3 (Li et al., 2014; García de la Cadena and Massieu, 2016; Zhang et al., 2018). In animal models of sepsis, increased markers of endoplasmic reticulum stress [e.g., glucose-regulated protein 94 (GRP94), CHOP, and caspase-12] have been observed in various organs including the heart and liver. Additionally, a direct correlation between these markers and the degree of organ dysfunction suggests that they may play a significant role in causing multi-organ failure in sepsis (Jiao et al., 2017). The induction of abnormal apoptosis due to endoplasmic reticulum stress in septic animals indicates that targeting endoplasmic reticulum stress-mediated apoptosis could be a promising avenue for research on clinical prevention and treatment strategies for sepsis. Traditional Chinese medicine has shown potential in attenuating lymphocyte apoptosis induced by sepsis through inhibition of the endoplasmic reticulum stress pathway (Xi et al., 2017). The characteristics of TCM extracts by the modulation of endoplasmic reticulum stres on sepsis are summarized in Table 4.

Genipin, derived from *Gardenia jasminoides* J.Ellis fruit, is a well-known traditional Chinese medicine with antipyretic and detoxifying properties. It has been demonstrated to possess anti-inflammatory, antioxidant, and bacteriostatic activities (Ishiguro et al., 1983; Luo et al., 2019). Recent studies have indicated that genipin can enhance the prognosis of sepsis while reducing sepsis-related liver damage and lung injury (Kim et al., 2012).

The impact of genipin on apoptosis induced by endoplasmic reticulum stress in spleen cells following CLP was further investigated (Luo et al., 2023). The findings demonstrated that genipin significantly attenuated the expression levels of splenic CHOP and caspase-3 proteins in CLP mice. Moreover, genipin markedly reduced the number of tunel-positive splenocytes. These results indicated that genipin possessed potential protective effects against sepsis. Furthermore, it was revealed that the underlying protective mechanism of genipin against sepsis involves inhibiting endoplasmic reticulum stress, downregulating CHOP protein expression, and mitigating splenocyte apoptosis.

Curcumin is a crucial active ingredient in *Curcuma longa* L. that exerts pharmacological effects. (Marchiani et al., 2014). Numerous animal experiments have consistently demonstrated that curcumin exhibits a broad spectrum of pharmacological activities including anti-inflammatory, anti-oxidative stress, anti-apoptotic, and immunomodulatory effects (Shehzad et al., 2010). Moreover, an extensive body of research has confirmed the protective effect of curcumin against sepsis and septic shock (Karimi et al., 2019; Wang S. et al., 2021; Vieira et al., 2023).

Resveratrol is a small non-flavonoidal polyphenolic compound present in various plants. Pharmacological studies have indicated that resveratrol exhibits diverse biological activities, such as anti-inflammatory, antioxidant properties, oxygen free radical scavenging abilities, and antitumor effects (Hiramatsu et al., 2015; Lu et al., 2018). Han et al. (2020) utilized the LPS method to induce the establishment of a rabbit model of acute lung injury (ALI). The rabbits were grouped and intervened with resveratrol and sodium chloride injection. The findings from this study demonstrated that resveratrol effectively reduced the protein level and mRNA expression of PERK, ATF4, CHOP, caspase-9, Bax while increasing the protein and mRNA expression level of Bcl-2 in ALI-induced lung tissues. Additionally, resveratrol attenuated histopathological injury induced by LPS in ALI rabbit lungs. These results suggest that resveratrol exerts a protective role against LPS-induced ALI in rabbits by mitigating endoplasmic reticulum stress and apoptosis.

The sepsis mouse model using the CLP method was successfully replicated by Huang et al. (2019b), and their experimental study demonstrated significant improvement in the overall condition of mice treated with ginsenoside Rb1 compared to those in the CLP group. Additionally, the alveolar tissues exhibited clear and intact structures with reduced infiltration of inflammatory cells, resulting in uniform-sized alveoli. Myocardial tissues showed decreased infiltration of inflammatory cells, visible transverse striations of the myocardium, and a reduction in necrotic cells. The expression levels of GRP78 and caspase-12 proteins in myocardial and lung tissues of the ginsenoside group exhibited a significant reduction, with highly statistically significant differences (*p* < 0.01). These findings suggest that ginsenoside Rb1 can attenuate the severity of infection and exert a protective effect on heart and lung tissue damage in septic mice. This mechanism may be attributed to the inhibition of excessive endoplasmic reticulum stress by ginsenoside Rb1.

Therefore, TCM extracts inhibit endoplasmic reticulum stress and reduce the expression of CHOP apoptotic protein, thereby alleviating the degree of cell apoptosis and achieving improvement in organ dysfunction caused by sepsis (show in Figure 2).

#### 4.4.3 Autophagy defect

Autophagy is a natural process in which cytoplasmic material or pathogens are phagocytosed and subsequently degraded through fusion with lysosomes. It serves as a crucial defense mechanism employed by the host to combat external pathogens and danger signals, while also playing an integral role in inducing and regulating the inflammatory response of innate immune cells - a key determinant influencing sepsis development (Qiu et al., 2019). Autophagy plays a protective role in sepsis, potentially through the following mechanisms: pathogen elimination, neutralization of microbial toxins, regulation of cytokine release, reduction of apoptotic cancer targets, and promotion of antigen expression (Nakahira et al., 2011; Ryter et al., 2014; Maurer et al., 2015). Chinese medicines can prevent sepsis by modulating autophagy-related signaling pathways, primarily involving the regulation of the AMPK pathway and PINK1/Parkin pathway. Additionally, they inhibit autophagy-related signaling pathways such as the JNK pathway, mTOR pathway, NF-κB pathway, as well as apoptosis to achieve anti-inflammatory effects and mitigate organ damage (Chang et al., 2023). The characteristics of TCM extracts by the modulation of autophagy defect on sepsis are summarized in Table 4.

Recently, there is mounting evidence indicating that autophagy defects may contribute to the development of sepsis-induced immunosuppression (Ren et al., 2017; Venet and Monneret, 2018). In a study, the expression of autophagy marker proteins LC3B and Beclin-l in sepsis-associated acute lung injury (ALI) was examined, along with the impact of allicin treatment on these markers (Peng et al., 2017). The findings revealed an increase in the expression of LC3B and Beclin-1 in lung tissues of mice with sepsis ALI, suggesting an upregulation in autophagy levels during septic ALI. Furthermore, treatment with allicin further augmented the expression of LC3B and Beclin-1 in lung tissue, indicating its ability to enhance autophagy levels and exert a protective effect against septic ALI. Another study showed that allicin enhanced the expression of LC3-B and Beclin-1 in hepatic tissues of septic mice, with a direct correlation to the level of autophagy (Gao et al., 2019). This study suggested that allicin’s hepatoprotective effect might be attributed to its modulation of autophagic activity.

The administration of artesunate upregulated autophagy and reversed the tolerant state in LPS-tolerant mice (Liu et al., 2020). Their findings unveiled a novel immunopharmacological effect of artesunate in reversing LPS tolerance by restoring autophagy. It has been demonstrated that artesunate restores cytokine production and enhances bacterial clearance through the induction of autophagy.

The mice were induced sepsis in through CLP and observed a decrease in hepatic expression of autophagy-associated protein Atg3, which was attenuated by genipin. CLP impaired autophagic flux, resulting in increased expression of hepatic microtubule-associated protein-1 light chain 3-II and sequestoome-1/p62 proteins; however, genipin restored the impaired autophagic flux and attenuated the CLP-induced decrease in hepatic lysosome-associated membrane protein-2 and Rab7 proteins as well as elevated expression of calpain 1 protein. These findings suggest that genipin protects against septic injury by restoring impaired autophagic flux through inhibition of the calpain system (Cho et al., 2016).

Xing et al. (2017) conducted *in vivo* and *in vitro* models of sepsis using CLP rats and LPS-treated human primary hepatocytes, respectively. They discovered that ginsenoside Rg3 can alleviates mitochondrial autophagy by activating the AMPK signaling pathway, increasing the levels of autophagy-associated proteins LC3BI, LC3II, and Beclin-1. The role of mitochondrial autophagy was investigated in rat liver and human primary hepatocytes. The effect of LPS on autophagy in mouse lung epithelial cells was observed a gradual increase in the expression of LC3II/LC3I (an autophagosome membrane protein) from 0 to 16 h followed by a decrease at 24 h (Ji et al., 2019). This suggested that autophagy initially increased but then decreased during the process induced by LPS. In addition, this study revealed that ginsenoside Rg1 administration increased autophagy and mitigated LPS-induced lung epithelial cell modulation (modulation rate decreased from 9.33% to 6.14%) after 24 h of LPS exposure in mouse lung epithelial cells, thereby attenuating sepsis-induced lung injury. Ginsenoside Rg1 was demostrated to protect against sepsis-induced brain injury by reducing the levels of autophagy-associated proteins LC3-II and p62 in the hippocampal region, inhibiting accumulation, and suppressing atypical Beclin-independent autophagy to safeguard against sepsis-induced brain injury (Li Y. et al., 2017). These studies collectively highlight the significance of modulating autophagy using ginsenosides within specific temporal and spatial parameters for effective sepsis treatment.

Traditional Chinese medicine extracts effectively modulate autophagy levels to mitigate sepsis, suppress inflammation, and attenuate multipe organ damage (show in Figure 2).

#### 4.4.4 Mitochondria damage

Mitochondria are crucial microcellular organelles involved in energy production, protein synthesis, and catabolism (Vringer and Tait, 2023). However, sepsis-induced mitochondrial damage or dysfunction can result in cellular damage, metabolic disorders, inadequate energy production, and oxidative stress. This ultimately leads to apoptosis of organ cells and immune cells, thereby causing multiorgan immune disorders, multiorgan failure, and even death. During sepsis, the antioxidant system mechanisms are disrupted due to limited oxygen supply and incomplete oxidative reactions as well as hypoxia. Consequently, there is a significant increase in the production of free radicals (Rocha et al., 2012). When exposed to DAMP or PAMP, activated leukocytes release inflammatory cytokines that stimulate NADPH-expressing oxidases (Quoilin et al., 2014). These cytokines induce an excessive production of reactive nitrogen species (RNS), which inhibits NO through the promotion of iNOS activity. The combination of NO and ROS can lead to peroxidation, resulting in the formation of RNS, which irreversibly inhibits electron transport chain (ETC.) activity. Consequently, this dysfunction of the ETC contributes to the generation of additional ROS within mitochondria during sepsis, unfortunately leading to further mitochondrial damage such as endosomal impairment, inhibition of ETC activity, and mitochondrial DNA damage (Singer, 2014). Eventually, the mitochondrial matrix undergoes swelling, leading to rupture of the mitochondrial membrane and initiation of apoptosis. Traditional Chinese medicine has the ability to modulate apoptosis balance, stabilize mitochondrial membrane potential, and regulate other pathways in order to exert a protective effect against sepsis-induced mitochondrial damage. The characteristics of TCM extracts by the modulation of mitochondria damage on sepsis are summarized in Table 4.

Nuclear related factor 2 (Nrf2) is the most potent transcription factor that regulates the antioxidant stress response in the organism. The stimulation of cells by oxygen free radicals leads to the phosphorylation of Nrf2, its dissociation from specific receptors, translocation to the nucleus, and subsequent initiation of the expression of various downstream antioxidant genes including HO-1 and NQO-1 (Liu et al., 2016; Senthil et al., 2016). The proteins HO-1 and NQO-1 possess antioxidant, anti-apoptotic, anti-inflammatory, and microcirculation maintenance functions, which play a crucial role in the body’s defense against oxidative stress (Choi, 2016; Hafez et al., 2019). The levels of Nrf2 were significantly reduced in sepsis-induced liver-injured rats, leading to an imbalance in the body’s oxidative/antioxidant system. This imbalance results in the release of a substantial amount of oxygen free radicals and subsequently induces oxidative stress injury in liver tissues (Yang et al., 2018). The alcoholic extract derived from *Hedysarum polybotrys* Hand.-Mazz. (*Radix hedysari*) was discovered to significantly enhance the phosphorylation level of pNrf2/Nrf2 and the expression levels of HO-1 and NQO-1 in hepatic tissues of septic rats, indicating that *R. hedysari* alcohol extract could elevate antioxidant activity and eliminate excessive oxygen free radicals in liver tissues of septic rats, thereby mitigating inflammatory damage to hepatic tissues (Yuan C. et al., 2021).

Aydin et al. (2016); Zhang et al. (2014) demonstrated that resveratrol exhibited a reduction in iNOS and NO concentrations, an increase in the ratio of reduced glutathione to oxidized glutathione (GSH/GSSG), as well as an elevation in peroxidase activity (catalase, superoxide dismutase, and other antioxidant enzymes). Moreover, these compounds effectively mitigated organ damage in septic mice.

Polydatin could effectively activate SIRT3, leading to the deacetylation of SOD2, reduction in ROS production, stabilization of endothelial adherens junction (AJ), and ultimately improved prognosis in septic mice through the protection of endothelial permeability (Wu J. et al., 2020). Polydatin enhanced the expression of CLS1 in SAKI through SIRT3, thereby promoting CL synthesis and improving mitochondrial function (Lei et al., 2022). *Astragalus* polysaccharides could enhance the energy metabolism of renal epithelial cells, improved the mitochondrial structure of the renal cortex, and mitigated histopathological changes in septic acute kidney injury rats (Wang X. et al., 2021).

The amide alkaloid ligustrazine was obtained through isolation and purification from the traditional Chinese medicine *Ligusticum chuanxiong* S.H.Qiu, Y.Q.Zeng, K.Y.Pan, Y.C.Tang & J.M.Xu. It acts as a novel calcium antagonist, exhibiting a wide range of pharmacological effects including antioxidant, anti-inflammatory, and antifibrotic activities (Ran et al., 2011). The compound can scavenge ROS by reducing plasma malondialdehyde (MDA) levels and increasing superoxide dismutase (SOD) activity, thereby providing protection against biofilm damage, acting as an antioxidant, and effectively improving myocardial injury in sepsis (Chen, 2011). Early administration of ligustrazine can effectively scavenge ROS, inhibit lipid peroxidation, and have other beneficial effects that contribute to improved patient prognosis (Wang, 2012; Liu, 2014). The Danhong injection exhibits a certain efficacy in combating myocardial damage induced by sepsis, as it effectively suppresses the levels of MDA and HMGB1 in the cardiac tissue of CLP septic rats while simultaneously enhancing SOD activity (Hu and Lou, 2016).

In summary, TCM extracts can exert a protective effect on sepsis-induced mitochondrial damage by inhibiting oxidative stress reactions, regulating the balance of oxidation/antioxidation, and eliminating ROS (show in Figure 2).

## 5 Discussion

As a significant form of complementary and integrated medicine, TCM possesses immense potential and advantages in the treatment of sepsis (Liang et al., 2015; Wang J. et al., 2022). The intervention of TCM can effectively leverage the role in addressing bacterial resistance during the diagnosis and treatment of sepsis patients, capitalizing on its distinct advantages across different stages. In the early stage of sepsis, the implementation of TCM as a complementary antibiotic therapy, particularly for patients with resistant bacterial infections, can effectively mitigate the emergence of drug-resistant bacteria, alter the resistance patterns exhibited by existing resistant or pan-resistant strains, and subsequently reduce the occurrence rate of multiple infections. The spleen and stomach play a crucial role in the development of sepsis by enhancing mental resilience and reducing the incidence of multiple organ dysfunction syndrome (MODS) through promoting clearance and minimizing turbidity. If MODS occurs, TCM can also be utilized to combat shock, dysfunction in blood coagulation, rectify the imbalance of inflammatory response, and mitigate organ damage. On the other hand, the exceptional merits of TCM are evident in its remarkable safety profile: according to a comprehensive investigation on drug-induced liver injury (DILI)-related adverse drug reactions (ADR) in China (L-ADR), Chinese medicine accounted for merely 4.5% of all reported ADR cases, while conventional drugs encompassing chemical and biological agents constituted 95.5%, with antibiotics including anti-tuberculosis medications representing the highest proportion (Wang J. et al., 2022).

In the treatment of sepsis and its complications, traditional Chinese medicine extracts and active ingredients have multiple effects such as anti-inflammatory properties, improvement of microcirculation, alleviation of gastrointestinal dysfunction, enhancement of the immune system, and prevention of organ damage. Traditional Chinese medicine extracts not only regulate the inflammation imbalance, improve impaired immune function, and enhance coagulation disorders to treat sepsis and improve patient prognosis. They also exert therapeutic effects on sepsis and multiple organ dysfunction by modulating the neuroendocrine-immune network, inhibiting endoplasmic reticulum stress, regulating autophagy defects, and improving mitochondrial damage. Given the complexity of chemical components in traditional Chinese medicine and the uncertainty of active ingredients, most studies have focused on isolating active compounds from prescriptions and single herbs for cellular or animal research. However, whether traditional Chinese medicine exacerbates multi-organ damage in sepsis patients due to potential side effects on relevant organs remains a crucial question that has not been thoroughly studied. Therefore, identifying and evaluating the potential harm of drugs on organ function is the main challenge faced by traditional Chinese medicine in terms of medication safety for sepsis treatment. In addition, there is currently no universally recognized standard for evaluating the efficacy of traditional Chinese medicine. Furthermore, the quality control of clinical trials for traditional Chinese medicine also needs improvement. In the future, a multidisciplinary collaborative effort will be necessary to elucidate the material basis of traditional Chinese medicine and provide important evidence for understanding the pathogenesis of sepsis and developing new drugs for treating sepsis. Therefore, it is imperative to introduce new systematic, standardized, and operationally strong guidelines in this research field to expedite the modernization and standardization process of traditional Chinese medicine.

## 6 Conclusion and future prospect

This review classifies and summarizes the efficacious monomeric constituents of traditional Chinese medicine for the treatment of sepsis based on their mechanisms of action. The findings demonstrate that the majority of potent monomeric components exert their effects by directly attenuating inflammatory factors, while some possess inhibitory properties against the NF-κB pathway or exhibit direct antagonism towards LPS. Others enhance patient survival rates by mitigating disseminated intravascular coagulation through anticoagulant activity. Furthermore, polysaccharides and saponins rectify functional abnormalities in sepsis via immunoregulatory effects, whereas specific monomers and extracts regulate autophagy to prevent and manage sepsis. This review provides valuable insights into the diverse pathways and therapeutic targets of monotherapy with TCM for sepsis treatment, facilitating a comprehensive comprehension of its underlying mechanisms.

The current research primarily focuses on the comprehensive study of TCM. The clinical application of TCM compound, such as Qingwenbaidu decoction (Leng et al., 2012), Dachengqi decoction, and Xuebijing injection (Ma and Song, 2009), is prevalent to therapy spesis; however, there is limited research on the TCM monomer. However, the mechanisms of action and target sites of TCM extracts are still unclear, and issues such as the lack of standardized dosages for effective ingredients from these components remain unresolved. The chemical structure of medicine, either similar to or belonging to the genus of TCM itself, exhibits a certain correlation. By means of animal and cell experimental research, it is possible to expedite the identification of TCM monomer compositions and leverage modern high-tech advancements for better understanding important targets and advantages in sepsis treatment. Therefore, the researchers can further facilitate the discovery or synthesis of analogs or derivatives, expanding the scope of the new drug research and development of TCM. The potential targets of TCM extracts and active components in the treatment of sepsis and its complications have been identified through a network pharmacological prediction, molecular docking analysis, and visual analysis in the field of molecular biology research. The experimental study on the mechanism of TCM treatment for sepsis, combined with the aforementioned methods, offers a novel direction in the quest for more efficacious intervention strategies. The research field requires the introduction of new systematic, normative, and operable guidelines to gain international recognition for the therapeutic advantages of TCM in treating acute and critical diseases. Most traditional Chinese medicines are administered orally, thereby entering the digestive tract. Consequently, the gut microbiota plays a pivotal role in the metabolism of these medications. Moreover, numerous traditional Chinese medicines exert an influence on the equilibrium of gut microbiota. Henceforth, future research endeavors should consider exploring the correlation between sepsis and gut microbiota status, as well as investigating the regulatory effects of traditional Chinese medicines on gut microbiota.

## Figures and Tables

**Table:** 

**TCM**	Traditional Chinese medicine
**DIC**	disseminated intravascular coagulation
**DAMP**	damage-associated molecular pattern
**PAMP**	pathogen-associated molecular pattern
**LPS**	lipopolysaccharide
**MTT**	methyl thiazolyl tetrazolium
**ELISA**	enzyme-linked immunosorbent assay
**TNF**	tumor necrosis factor
**IL**	interleukin
**CLP**	cecal ligation and puncture
**TLR-4**	Toll-like receptor 4
**SphK1**	sphingosine kinase 1
**ERK1/2**	extracellular signal-regulated kinase 1/2
**NF**	nuclear factor
**HMGB1**	high mobility group protein B1
**APS**	*Astragalus* Polysaccharides
**iNOS**	inducible nitric-oxide synthase
**COX-2**	cyclooxygenase-2
**PGE2**	prostaglandin E2
**ICAM-1**	intercellular adhesion molecule-1
**VCAM-1**	vascular cell adhesion molecule-1
**IRF4**	interferon regulatory factor 4
**MUM1**	multiple myeloma oncogene 1
**NLRP**	nucleotide-binding domain, leucine-rich repeat, and pyrin domain-containing protein
**TGF-β**	transforming growth factor-β
**Th**	T lymphocyte subgroup
**Treg**	regulatory T cell
**JAK2**	Protein tyrosine kinase 2
**STAT3**	Signal transducers and activator 3
**IFN**	interferon
**MAPK**	mitogen activated protein kinase
**ERK**	extracellular signal regulated kinase
**TF**	tissue factor
**PT**	prothrombin time
**APTT**	activated partial thromboplastin time
**THR**	threonine
**EPCR**	endothelial cell protein c receptor
**TACE**	tumor necrosis factor-a converting enzyme
**FIB**	fibrinogen
**CAP**	cholinergic anti-inflammatory pathway
**ACh**	acetylcholine
**α7nAChR**	α7 nicotinic ACh receptors
**ChAT**	choline acetyltransferase
**AChE**	acetylcholinesterase
**LRP1**	lipoprotein receptor-related protein 1
**CHOP**	C/EBP homologous protein
**PERK**	pkr-like endoplasmic reticulum kinase
**IRE1**	inositol-requiring enzyme 1
**HLA-DR**	human leukocyte antigen-DR
**ATF6**	transcriptional activator 6
**RNS**	reactive nitrogen species
**Nrf2**	nuclear related factor 2
**ROS**	reactive oxygen species
**ETC.**	electron transport chain
**MDA**	malondialdehyde
**HO-1**	Hemeoxygenase-1
**NQO-1**	ubiquinone oxidoreductase
**LC**	light chain
**PINK 1**	PTEN induced putative kinase 1.
